# SAME TACO: Making the Philosophy of Realist Evaluation Accessible to Healthcare Professionals

**DOI:** 10.1111/jep.70742

**Published:** 2026-11-09

**Authors:** Alejandro Argüelles Bullón

**Affiliations:** ^1^ Division of Health Research, Faculty of Health and Medicine Lancaster University Lancaster UK

## 1 Introduction

Realist evaluation is increasingly being used across healthcare and evaluation to understand how and why interventions work, for whom, and under what circumstances [1]. Realist approaches have contributed to healthcare evaluation both through their philosophical foundations for understanding complex interventions [2] and their practical application to understanding how and why healthcare practices are implemented and sustained [3]. However, while realist approaches offer exciting opportunities for healthcare professionals, learning to think like a realist can sometimes feel overwhelming. For those new to realist evaluation, the challenge is not necessarily understanding its value but engaging with the philosophical foundations that underpin it [4, 5, 6]. Commonly used jargon such as stratified ontology, generative causation, emergence, agency, demi‐regularities and others can be a barrier towards using and implementing realist approaches. Realist researchers have invested considerable effort in making realist evaluation a part of the healthcare research repertoire, but have we paid enough attention to making its philosophical foundations accessible to those expected to use it? Although work has been undertaken to explain and operationalise realist approaches [7, 8], the terminology and philosophical arguments can still make them feel inaccessible, particularly for busy healthcare professionals without a background in philosophy.

Having learned, applied and taught realist approaches, I have continually asked myself how we can make realist philosophy engaging and memorable without losing the very ideas that make it distinctive. After all, realist evaluation is not simply about following a methodological recipe, but it is underpinned by a particular way of understanding how the world works and how we can develop explanations about it. Inspired by Curran's [9] work on making implementation science accessible through a simple teaching slide, I set myself a challenge. Could I capture the key philosophical ideas underpinning realist thinking in one teaching slide? The aim was not to replace philosophical depth with simplicity but to provide an accessible starting point from which healthcare professionals could begin to remember and apply these complex ideas.

## 2 SAME TACO: Eight Ideas of Realist Thinking

SAME TACO is a mnemonic bringing together eight important philosophical ideas that can help learners begin to think like realists: Stratified ontology, Agency and structure, Mechanisms, Emergence, Tendencies and demi‐regularities, Absence, Context and Open systems (Figure 1). The mnemonic is not intended to resolve the philosophical debates surrounding these concepts or provide an exhaustive account of realist philosophy. At its core, it offers educators a memorable way to introduce them and healthcare professionals a starting point for further exploration.

**Figure 1:**
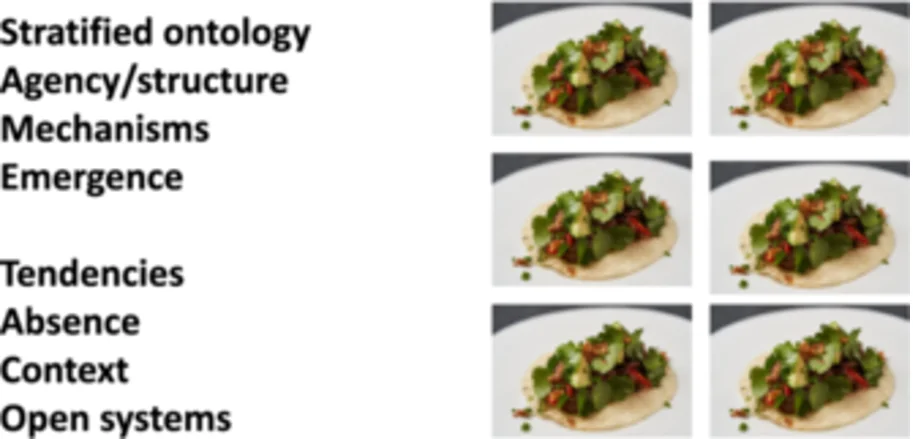
SAME TACO: A mnemonic illustrating eight key philosophical ideas underpinning realist thinking.

Each idea is briefly introduced in one or two sentences, accompanied by a familiar healthcare example that can be tailored to different audiences. The intention is not to explore the philosophical depth surrounding each concept, as these have been extensively discussed elsewhere [10, 11, 12, 13], but rather to provide an accessible starting point for understanding the key ideas underpinning realist thinking. To illustrate these concepts, we use a brief example of an intervention involving follow‐up telephone calls to patients after hospital discharge.

S—Stratified ontology. Reality involves more than what we can directly observe or experience. Realists distinguish between the empirical (what is experienced), the actual (what happens) and the real (underlying causal powers and mechanisms) [14]. Understanding whether patients received follow‐up calls is therefore only part of the story; we also want to understand what made those calls meaningful or ineffective.

A—Agency and structure. People's actions are shaped, but not entirely determined, by the social and organisational structures surrounding them. Equally, people can reproduce or transform those structures through their actions [15]. For example, staffing policies may constrain nurses’ ability to deliver follow‐up care while nurses may adapt existing routines to better support patients.

M—Mechanisms. Realists seek to explain outcomes through underlying causal processes rather than simply observing associations between interventions and outcomes. Mechanisms have been described as hidden, context‐sensitive causal forces as well as how people respond to the resources and opportunities an intervention provides [16, 17]. A follow‐up call might encourage patients to seek help because it provides reassurance and increases their confidence rather than because the call itself automatically produces improvement.

E—Emergence. The whole cannot always be understood by examining its individual parts. Interactions between people, organisations and processes can generate new properties and outcomes [18]. For example, continuity of care may emerge through interactions between patients and healthcare professionals rather than from any single activity.

T—Tendencies and demi‐regularities. In open systems, outcomes may demonstrate recurring but imperfect patterns rather than universal regularities [19]. For example, follow‐up calls may improve patient engagement in some circumstances but not others. These patterns provide clues for investigating the mechanisms and contexts responsible.

A—Absence. What is missing can be as important as what is present. An expected event may not occur because a necessary condition or resource is absent [20]. For example, the absence of interpreting support may prevent a follow‐up call from generating reassurance even when the call takes place.

C—Context. Context comprises the layered, changing conditions that enable or shape how mechanisms operate and outcomes arise [21]. For example, organisational culture and patients’ social circumstances may all influence how follow‐up care works.

O—Open systems. Healthcare operates within dynamic systems where multiple causal influences interact and conditions continually change [22]. For example, staffing pressures and organisational priorities may simultaneously affect the delivery and outcomes of follow‐up care.

Collectively, these eight ideas encourage learners to look beyond whether an intervention works and begin asking deeper questions about causation, human action, structure, context and complexity. The slide can be used as a starting point for discussing how these philosophical ideas inform programme theory development and explanatory evaluation in healthcare research.

## 3 Making Realist Thinking Stick

I have used SAME TACO when teaching realist approaches to differentaudiences, including healthcare professionals and experienced realist researchers, in Canada, India and the UK with a further presentation scheduled in New Zealand for November 2026. The informal feedback has been encouraging with several participants describing the slide as a moment when previously difficult concepts began to make sense. One memorable reaction was: “I will never think of a TACO the SAME way again.” Although these experiences are anecdotal rather than evidence of teaching effectiveness, they have reinforced my belief that making philosophical concepts memorable can help create opportunities for deeper learning. When learners can remember the fundamental ideas, they may be better positioned to question their assumptions about causation and apply realist thinking to practical healthcare problems. For healthcare evaluation, this matters. Its explanatory value depends on researchers understanding why mechanisms and contexts matter and why outcomes cannot always be explained through linear relationships. The philosophy should inform the evaluation rather than simply appear in its introduction.

SAME TACO is not perfect, nor does it attempt to capture every aspect of realist philosophy. Important concepts such as retroduction and judgemental rationality are not explicitly represented, while some of the eight ideas draw from broader critical realist traditions that are not emphasised equally across all realist approaches. Furthermore, the mnemonic does not eliminate the philosophical jargon that can make realist approaches difficult to understand. Its accessibility lies in bringing these concepts together in one memorable slide, providing a starting point from which each idea can be explained and illustrated using examples relevant to learners’ professional contexts. Beyond its teaching value, SAME TACO may also serve as a simple philosophical guide for those undertaking realist evaluations, prompting researchers to reflect on the underlying assumptions informing their methodological decisions and philosophical principles they should demonstrate throughout their work. However, it is not a substitute for deeper engagement with foundational literature or methodological guidance [23, 24]. Its value lies instead in providing an accessible entry point. The slide works best when accompanied by explanation and examples relevant to learners’ professional contexts. Future work could explore whether using the mnemonic improves learners’ understanding and application of realist concepts, including across different healthcare disciplines and educational settings.

## 4 Conclusion

Realist evaluation offers healthcare professionals valuable ways to understand complexity and develop explanations about how interventions work. Yet its philosophical foundations can remain intimidating for those encountering them for the first time. I developed SAME TACO to make these foundations more accessible and memorable to teach and learn. It is not intended to simplify away the richness of realist philosophy, but to help people begin engaging with it. If we want realist approaches to become more accessible across healthcare, we need to pay attention not only to how we conduct evaluations, but also to how we introduce the thinking behind them.

## Author Contribution

Alejandro Argüelles Bullón is the sole author of this manuscript and is responsible for its conception, development, drafting, revision and final approval.

## Funding

The author has nothing to report.

## Ethics Statement

Ethics approval was not required, as this article does not report original empirical research involving human participants, animals or identifiable personal data.

## Consent

Patient consent was not required, as this article does not report patient‐level data or original empirical research involving human participants.

## Conflicts of Interest

The author declares no conflicts of interest.

## Permission to Reproduce Material From Other Sources

Figure 1 presents an original slide developed by Alejandro Argüelles Bullón, the author of this manuscript, who retains ownership of the original work. No third‐party copyright permissions are required for its reproduction.

## Data Availability

The author has nothing to report.
